# Is substance use associated with HIV cascade outcomes in Latin America?

**DOI:** 10.1371/journal.pone.0194228

**Published:** 2018-03-15

**Authors:** Raquel B. De Boni, Meridith B. Peratikos, Bryan E. Shepherd, Beatriz Grinsztejn, Claudia Cortés, Denis Padgett, Eduardo Gotuzzo, Pablo F. Belaunzarán-Zamudio, Peter F. Rebeiro, Stephany N. Duda, Catherine C. McGowan, for CCASAnet

**Affiliations:** 1 Instituto Nacional de Infectologia Evandro Chagas- FIOCRUZ, Rio de Janeiro, Brazil; 2 Vanderbilt University Medical Center, Nashville, TN, United States of America; 3 Fundación Arriaran–Facultad de Medicina Universidad de Chile, Santiago, Chile; 4 Instituto Hondureno de Seguridad Social and Hospital Escuela, Tegucigalpa, Honduras; 5 Universidad Peruana Cayetano Heredia, Lima, Peru; 6 Departamento de Infectología, Instituto Nacional de Ciencias Médicas y Nutrición Salvador Zubirán, Mexico City, Mexico; National and Kapodistrian University of Athens, GREECE

## Abstract

**Background:**

The HIV care cascade has improved in Latin America over the last decade. However, the influence of alcohol and noninjected drug use (NIDU) on cascade outcomes is mostly unknown. This study estimated the association of alcohol and NIDU with retention in care, loss to follow up (LTFU), and virologic failure (VF).

**Methods:**

Individuals ≥18 years attending routine HIV clinic visits and completing the Rapid Screening Tool (RST; evaluating NIDU and ART adherence in 7-day recall period) during 2012–13 were followed up to 2015 in the Caribbean, Central and South America network for HIV epidemiology. Adjusted odds ratios (aOR) were calculated for the association of alcohol consumption and NIDU with retention in care by logistic regression; adjusted hazard ratios (aHR) were estimated for the associations with LTFU and VF by Cox regression.

**Results:**

Among 3604 individuals, the proportions retained in care for one year were 84%, 79%, 72%, and 69% for patients reporting non-use, alcohol use, NIDU, and both alcohol and NIDU, respectively. For the same patient groups, the proportions LTFU over 18 months were 6%, 8%, 12%, and 13%, respectively. There were 1901 patients (53%) with HIV RNA results; VF proportions were similar between users and nonusers (ranging from 14–16%). After controlling for age, sex, study site, HIV transmission mode, time on ART, AIDS status, and CD4 count, neither alcohol use (aOR = 1.1, CI = 0.9–1.4; aHR = 1.0, CI = 0.8–1.3) nor NIDU (aOR = 1.3, CI = 0.9–1.8; aHR = 1.4, CI = 0.9–2.1) were significantly associated with retention or VF, respectively. However, both alcohol use (aHR = 1.2, CI = 1.02–1.4) and NIDU (aHR = 1.3, CI = 1.00–1.8) were associated with increased LTFU.

**Conclusion:**

Alcohol use and NIDU in a 7-day recall period increased the risk of being LTFU during the next 18 months, highlighting the need for routine screening and targeted interventions to keep these individuals in care and on ART.

## Introduction

The HIV cascade of care [1,2,3] is a powerful framework to describe and monitor the complex and dynamic process of HIV infection treatment using well-defined, specific stages of care. The main stages, or outcomes, of the care cascade are HIV diagnosis, linkage and retention in care, combination antiretroviral therapy (cART) use, and viral suppression. As a strategy to end the HIV epidemic, in 2014, the Joint United Nations Programme on HIV/AIDS (UNAIDS) established the goal to have 90% of persons living with HIV/AIDS (PLWHA) aware of their HIV status, 90% of those diagnosed receiving cART, and 90% of those on cART virally suppressed by 2020 [4]. Understanding, monitoring, and intervening on factors that may prevent this goal’s accomplishment are public health priorities. Among individuals who were already diagnosed and linked to care in Latin America, retention, cART use and viral suppression significantly improved from 2003 to 2012 (63 to 77%, 74 to 91% and 53 to 82%, respectively) [5].

Since the pioneering work of Singer [6], HIV and substance use (SU) are considered syndemic conditions (i.e., conditions that may cluster and act synergistically producing worse health outcomes [7]), and it is possible that PLWHA and SU present worst outcomes in the HIV cascade of care. A qualitative review among substance users in the United States concluded that SU had worse outcomes at all points along the cascade of care [8]. Alcohol and noninjected drug use (NIDU) were associated with lower retention in care [9,10] and a history of NIDU decreased the chance of returning to care after a treatment gap [11]. This is particularly important because retention/engagement in care is critical to starting cART and to achieving viral suppression [12]. In addition, alcohol and NIDU have been associated with lack of adherence (a major factor for VF) and worse clinical outcomes among PLWHA [8,13].

The influence of alcohol use and NIDU on care cascade outcomes has not been studied in Latin America. Both HIV and NIDU present with different epidemiologic profiles across the globe. In many Latin American countries, HIV is a concentrated epidemic where new cases cluster among key-populations, especially men who have sex with men, transgender women, sex workers, and substance users [14]. UNAIDS estimates that cART coverage in the region is 55% (47–64%) with about 1.1 million people on cART; a substantial increase in cART coverage over the last decade is thought to be the main factor behind decreasing rates of AIDS mortality [15]. Considering data from the general population in the region, the consumption of alcohol was estimated at 7.5–9.9 liters of pure alcohol per capita while the prevalence of 12-month abstention was at 40–60% in 2010 [16]. Regarding NIDU, cannabis is the most frequent substance used, and there has been an increase in cocaine use over the past few years [17]. The proportion of persons treated for substance use in the region, however, remains low. The highest numbers of individuals in treatment are being treated for cocaine use disorders, but treatment for cannabis use disorders seems to be increasing [17].

The Caribbean, Central and South America network for HIV epidemiology (CCASAnet) is one of seven member regions of the NIH-funded International epidemiology Databases to Evaluate AIDS (IeDEA) consortium [18]. Factors associated with HIV cascade outcomes between 2003 and 2012 were evaluated in this cohort [5]. It was found that female sex and injecting drug use as HIV transmission mode were significantly associated with lower retention in care, but unrelated to cART use or viral suppression. In 2012, a Rapid Screening Tool (RST) was implemented at six CCASAnet sites evaluating self-reported 7-day recall of alcohol and NIDU; cross-sectional analysis found that both increased the chance of self-reporting missed cART doses [19]. The present study aims to expand the understanding of these two interconnected epidemics in Latin America benefiting from CCASAnet data to estimate the association of alcohol and NIDU with three HIV cascade outcomes: 1) retention in care, 2) loss to follow up (LTFU) and 3) virologic failure (VF).

## Methods

### Study design and population

A prospective study was conducted after the RST implementation in CCASAnet. The CCASAnet consortium and implementation of the RST are detailed elsewhere [19]. In brief, CCASAnet (www.CCASAnet.org) is a dynamic clinical HIV cohort established in 2006 that brings together data from seven Latin American countries [18]. We herein analyzed data from HIV-positive adults (≥ 18 years of age) on cART, who completed the RST when attending routine clinical visits at CCASAnet sites between June 11, 2012 and December 13, 2013. These individuals were then followed until May 1st, 2015. Sites contributing data were Hospital Fernández, Buenos Aires, Argentina (FH-Argentina); Instituto Nacional de Infectologia Evandro Chagas, FIOCRUZ, Rio de Janeiro, Brazil (INI- Brazil); Fundacion Arriaran, Santiago, Chile (FA-Chile); Instituto Hondureno de Seguridad Social and Hospital Escuela, Tegucigalpa, Honduras (IHSS/HE-Honduras); Instituto Nacional de Ciencias Medicas y Nutricion Salvador Zubiran, Mexico City, Mexico (INCMNSZ-Mexico); and Instituto de Medicina Tropical Alexander von Humboldt, Lima, Peru (IMTAvH-Peru). Institutional review boards at each aforementioned site and at the Vanderbilt University approved the study.

### Outcomes

Retention in care was defined as 2 or more HIV care visits at least 90 days apart (as recommended by the US Institute of Medicine[20]), during the year defined by May 1, 2014 through May 1, 2015.

Loss to follow-up (LTFU) was defined retrospectively as no contact for at least 1 year before database close (May 1^st^, 2015). This definition allows individuals with large gaps in care to return to the cohort. Sites in Brazil, Chile, Mexico and Peru reviewed national death registries to trace LTFU patients for mortality.

Virologic failure was defined as one of the following: (1) two consecutive HIV-1 RNA measurements above 50 copies/mL; (2) a single HIV-1 RNA measurement of above 1000 copies/mL [21].

### Measures and definitions

#### Primary exposure variables

Alcohol and NIDU were captured with the RST and two variables were created based on self-report: 1) any alcohol consumption in the past 7 days and 2) any NIDU (marijuana, cocaine, crack, or heroin) in the past 7 days. If multiple questionnaires were collected between June 11, 2012 and December 13, 2013, only the most recent questionnaire was used [19].

#### Covariates

Information regarding follow-up, age, sex, HIV transmission mode, AIDS status prior to cART initiation (pre-cART AIDS), CD4 counts (prior to ART and at time of RST using a window of ±6 months), and time on cART (at time of RST) were retrieved from the CCASAnet clinical cohort dataset (updated to May 2015), in accordance with the CCASAnet Standard Procedure for Data Transfer.

Adherence: Individuals who answered that they forgot one or more pills in a 7-day recall period in the RST were considered non-adherent.

In addition, we described all-cause mortality up to May 2015 (retrieved from the CCASAnet dataset, as detailed by Carriquiry et al. [22]). Basic site characteristics regarding the provision of substance use treatment were retrieved from the IeDEA Site Assessment 2.0, which was collected in 2014 and summarizes the availability of services at various CCASAnet sites [23].

### Statistical analysis

Outcomes and covariates were described by alcohol and NIDU; frequencies were compared using chi-square tests, and continuous variables were compared using Kruskal-Wallis tests. The association between alcohol and NIDU with retention in care was estimated using multivariable logistic regression, adjusting for all covariates listed above. Patients who died or transferred prior to May 1, 2015 were excluded from this model. We also conducted secondary analyses using the same covariates but including alcohol as a continuous variable (number of drinks in last week) and as a categorical variable (No drinks, 1–7 drinks/week, 8–14 drinks/week, >14 drinks/week).

Cumulative incidence of LTFU was stratified by alcohol use and NIDU, and calculated by treating death as a competing risk; Gray's test was used to test the null hypothesis of no association between alcohol/NIDU and LTFU. The association among alcohol and NIDU with LTFU was estimated using cause-specific hazard ratios in multivariable Cox regression stratified by site and controlling for all covariates. Patients who died or transferred were censored in the Cox model. Patients who were LTFU were considered to have the event at the date they were last known to be alive.

Cumulative incidence of VF was stratified by alcohol and NIDU and calculated by treating death as a competing risk; Gray's test was used to test the null hypothesis of no association between alcohol/NIDU and VF. Patients lost to follow-up were censored at their last visit. Using multivariable Cox regression stratified by site, we estimated the associations between alcohol/NIDU and time to VF adjusting for the covariates listed above.

Multiple imputation was used to account for missing covariates. The functions "aregImpute" and "fit.mult.impute" from the Hmisc package in R were used. These packages use predictive mean matching to take random draws from imputation models; 25 imputation data sets were used. Linearity assumptions for continuous variables were examined and restricted cubic spline transformations were used where appropriate. For retention in care, LTFU, and VF, secondary models including self-reported adherence were fit so that we could examine the influence of substance use on these outcomes independent of adherence behavior. In addition, these models were useful for exploring possible associations between adherence (measured by a single yes/no question) and retention in care and LTFU. Interactions between alcohol and NIDU were tested in all models.

R version 3.2.5 (2016-04-14) (www.r-project.org) was used for data analyses. Analysis code is posted at biostat.mc.vanderbilt.edu/ArchivedAnalyses.

## Results

The sample was comprised of 3604 individuals receiving care for HIV in the six CCASAnet sites. Most patients were male (n = 2739, 76%) and the median age was 42 years (interquartile range, IQR, 34–50 years). Overall, 26% reported having at least one alcoholic drink but no NIDU in a 7-day recall period, 2% reported NIDU only, and 3.5% reported both alcohol and NIDU. The most frequent NIDU used was cannabis (n = 169), followed by cocaine (n = 57). Patient characteristics, including patient outcomes, both overall and by alcohol/NIDU category are reported in Table 1.

**Table 1:** Outcomes and characteristics of the CCASAnet study population by substance use, 2012–2015.

	None	Alcohol use only	NIDU only	Alcohol and NIDU	Total	P-value
	(n = 2457)	(n = 944)	(n = 76)	(n = 127)	(n = 3604)	
**Outcomes**						
Retained in care for 1 year	2056 (84%)	749 (79%)	55 (72%)	87 (69%)	2947 (82%)	< 0.001
LTFU in 18 months, n(%)	150 (6%)	76 (8%)	9 (12%)	17 (13%)	252 (7%)	0.002
VF in 18 months, n(%)	187 (16%)	79 (14%)	6 (14%)	13 (16%)	285 (15%)	0.70
Missing VF data	1259 (51%)	363 (38%)	33 (43%)	48 (38%)	1703 (47%)	
**Demographic covariates**						
Female	708 (29%)	137 (15%)	9 (12%)	11 (9%)	865 (24%)	< 0.001
Age (years), median (IQR)	43 (35–50)	41 (34–49)	39 (30–46)	36 (31–44)	42 (34–50)	< 0.001
Age group, n(%)						< 0.001
18–29 years	279 (11%)	128 (14%)	18 (24%)	26 (20%)	451 (13%)	
30–39 years	738 (30%)	303 (32%)	24 (32%)	55 (43%)	1120 (31%)	
40–49 years	805 (33%)	308 (33%)	23 (30%)	38 (30%)	1174 (33%)	
≥ 50 years	635 (26%)	205 (22%)	11 (14%)	8 (6%)	859 (24%)	
Site, n(%)						< 0.001
Argentina	183 (7%)	91 (10%)	20 (26%)	32 (25%)	326 (9%)	
Brazil	962 (39%)	329 (35%)	24 (32%)	22 (17%)	1337 (37%)	
Chile	337 (14%)	350 (37%)	16 (21%)	37 (29%)	740 (21%)	
Honduras	188 (8%)	68 (7%)	5 (7%)	20 (16%)	281 (8%)	
Mexico	702 (29%)	63 (7%)	9 (12%)	14 (11%)	788 (22%)	
Peru	85 (3%)	43 (5%)	2 (3%)	2 (2%)	132 (4%)	
HIV transmission mode, n(%)						< 0.001
Heterosexual contact	1066 (43%)	300 (32%)	24 (32%)	24 (19%)	1414 (39%)	
Homosexual contact	1135 (46%)	561 (59%)	44 (58%)	83 (65%)	1823 (51%)	
Injecting drug user	25 (1%)	5 (1%)	1 (1%)	4 (3%)	35 (1%)	
Other/unknown	231 (9%)	78 (8%)	7 (9%)	16 (13%)	332 (9%)	
**Clinical covariates**						
CD4 count at cART initiation, median (IQR)	194 (77–317)	201 (85–312)	220 (122–325)	200 (92–290)	197 (79–316)	0.75
Missing, n(%)	300 (12%)	118 (12%)	13 (17%)	20 (16%)	451 (13%)	
CD4 count at cART initiation, n(%)						0.85
< 350 cells/mm^3^	1732 (80%)	666 (81%)	48 (76%)	85 (79%)	2531 (80%)	
≥ 350 cells/mm^3^	425 (20%)	160 (19%)	15 (24%)	22 (21%)	622 (20%)	
CD4 count at RST administration, median (IQR)	500 (319–690)	489 (334–699)	504 (288–820)	482 (311–688)	495 (321–692)	0.94
Missing, n(%)	179 (7%)	92 (10%)	13 (17%)	17 (13%)	301 (8%)	
CD4 count at RST administration, n(%)						0.61
< 350 cells/mm^3^	648 (28%)	224 (26%)	19 (30%)	33 (30%)	924 (28%)	
≥ 350 cells/mm^3^	1630 (72%)	628 (74%)	44 (70%)	77 (70%)	2379 (72%)	
pre-cART AIDS status, n(%)						0.28
No	689 (28%)	278 (29%)	20 (26%)	31 (24%)	1018 (28%)	
Yes	1375 (56%)	495 (52%)	38 (50%)	73 (57%)	1981 (55%)	
Unknown	393 (16%)	171 (18%)	18 (24%)	23 (18%)	605 (17%)	
cART initiation to RST administration (years), median (IQR)	5 (2–10)	5 (2–9)	6 (2–9)	4 (2–8)	5 (2–9)	0.077
Missed ART dose in 7-day recall, n(%)						< 0.001
No	2184 (89%)	682 (72%)	49 (64%)	67 (53%)	2982 (83%)	
Yes	273 (11%)	262 (28%)	27 (36%)	60 (47%)	622 (17%)	
**Detailed substance use**						
Alcohol, n(%)						< 0.001
No drinks	2457 (100%)	0 (0%)	76 (100%)	0 (0%)	2533 (70%)	
1–3 drinks	0 (0%)	521 (55%)	0 (0%)	52 (41%)	573 (16%)	
> 3 drinks	0 (0%)	423 (45%)	0 (0%)	75 (59%)	498 (14%)	
Marijuana, n(%)						< 0.001
No	2457 (100%)	944 (100%)	7 (9%)	27 (21%)	3435 (95%)	
Yes	0 (0%)	0 (0%)	69 (91%)	100 (79%)	169 (5%)	
Cocaine, n(%)						< 0.001
Missing	0 (0%)	0 (0%)	0 (0%)	1 (1%)	1 < 1%)	
No	2457 (100%)	944 (100%)	65 (86%)	80 (63%)	3546 (98%)	
Yes	0 (0%)	0 (0%)	11 (14%)	46 (37%)	57 (2%)	
Crack, n(%)						< 0.001
No	2457 (100%)	944 (100%)	72 (95%)	119 (94%)	3592 (100%)	
Yes	0 (0%)	0 (0%)	4 (5%)	8 (6%)	12 < 1%)	
Heroin, n(%)						< 0.001
No	2457 (100%)	944 (100%)	74 (97%)	127 (100%)	3602 (100%)	
Yes	0 (0%)	0 (0%)	2 (3%)	0 (0%)	2 < 1%)	
Any NIDU, n(%)						< 0.001
No	2457 (100%)	944 (100%)	0 (0%)	0 (0%)	3401 (94%)	
Yes	0 (0%)	0 (0%)	76 (100%)	127 (100%)	203 (6%)	

NIDU = noninjected drug use; LTFU = Lost to follow-up; VF = virological failure; IQR: Interquartile Range; RST = Rapid Screening Tool.

A total of 657 individuals were not retained in care (18%, n = 657/3549). Retention was 84% among those who did not report any alcohol or NIDU, 79% among those reporting alcohol use only, 72% among those reporting NIDU only, and 69% among those reporting both alcohol and NIDU. Most who were not retained were male (82%) and median age was 40 years (IQR 34–48). Table 2 shows the association between patient characteristics and the odds of not being retained in care after one year of follow up. As the outcome was not rare in this population, the odds do not approximate risks, though they are still valid measures of the strengths of association. After adjusting for patient characteristics, neither alcohol use nor NIDU were statistically associated with retention in care (adjusted odds ratio [aOR] = 1.11, 95% confidence interval [CI] 0.90–1.38; aOR = 1.26, 95% CI 0.88–1.80, respectively). Results were similar when self-reported adherence was included in the model. Younger individuals were more likely to not be retained (e.g., for 20- versus 30-year-olds, aOR = 1.23, 95% CI 1.11–1.36). Other factors that were associated with increased odds of not being retained in care were male sex, longer time on ART, and study site.

**Table 2:** Logistic regression models to estimate factors associated with not being retained in care at CCASAnet sites during May 1, 2014 to May 1, 2015, not controlling and controlling for adherence (n = 3549)^a^^,^^b^^,^^c^^,^^d^.

	Model omitting adherence		Model including adherence	
	aOR (95% CI)	p-value	aOR (95% CI)	p-value
**Primary Exposures**				
Alcohol use	1.11 (0.90, 1.38)	0.33	1.07 (0.86, 1.33)	0.56
NIDU	1.26 (0.88, 1.80)	0.20	1.19 (0.83, 1.71)	0.33
**Demographic covariates**				
Age		< 0.001		< 0.001
20 years	1.23 (1.11, 1.36)		1.23 (1.11, 1.36)	
30 years (ref)	1		1	
40 years	0.81 (0.73, 0.90)		0.81 (0.74, 0.90)	
50 years	0.66 (0.54, 0.81)		0.66 (0.54, 0.81)	
Male (vs. Female)	1.35 (1.00, 1.81)	0.051	1.34 (0.99, 1.80)	0.057
Site		< 0.001		< 0.001
Brazil (ref)	1		1	
Argentina	15.67 (10.82, 22.68)		15.81 (10.91, 22.89)	
Chile	8.00 (5.67, 11.27)		7.96 (5.65, 11.22)	
Honduras	16.34 (11.05, 24.16)		15.88 (10.72, 23.52)	
Mexico	5.06 (3.55, 7.22)		5.23 (3.66, 7.48)	
Peru	1.32 (0.58, 3.00)		1.31 (0.58, 2.97)	
HIV transmission mode		0.68		0.73
Heterosexual (ref)	1		1	
Homosexual	1.17 (0.90, 1.52)		1.16 (0.89, 1.51)	
IDU	1.05 (0.41, 2.72)		1.03 (0.40, 2.67)	
Other/unknown	1.16 (0.81, 1.67)		1.14 (0.79, 1.63)	
**Clinical covariates**				
pre-cART AIDS	0.82 (0.61, 1.10)	0.19	0.83 (0.62, 1.12)	0.22
CD4 at cART initiation (per 100 cells/mm^3^)	0.94 (0.85, 1.03)	0.17	0.94 (0.85, 1.03)	0.16
CD4 at RST administration (per 100 cells/mm^3^)	0.95 (0.91, 0.99)	0.026	0.95 (0.91, 1.00)	0.039
Time on cART (per 1 year increase)	1.03 (1.01, 1.06)	0.012	1.03 (1.01, 1.06)	0.013
Missed cART dose(s) in 7-day recall	-	-	1.26 (0.98, 1.63)	0.076

a] Retention is measured by 2 or more visits at least 90 days apart during the period of interest (May 1, 2014 to May 1, 2015).

b] There was little evidence of non-linearity for all continuous covariates with the log-odds of retention.

c] There are 3549 observations included in this model. 55 deaths were excluded.

d] There is little evidence of an interaction between alcohol and drug use on the log-odds of retention (p = 0.54).

Adjusting for adherence, there is little evidence of an interaction between alcohol and drug use on the log-odds of retention (p = 0.50). Interaction terms were excluded from the models summarized above. NIDU = noninjected drug use; RST = Rapid Screening Tool.

In a secondary analysis controlling for the same covariates, a higher number of alcoholic drinks increased the chance of not being retained in care (aOR = 1.19, 95% CI 1.08–1.30 per 7-drink increase; and aOR = 2.54, 95% CI 1.35–4.78 for >14 drinks compared to no drinks in 7-day recall period; see S1 Table).

**Fig 1:**
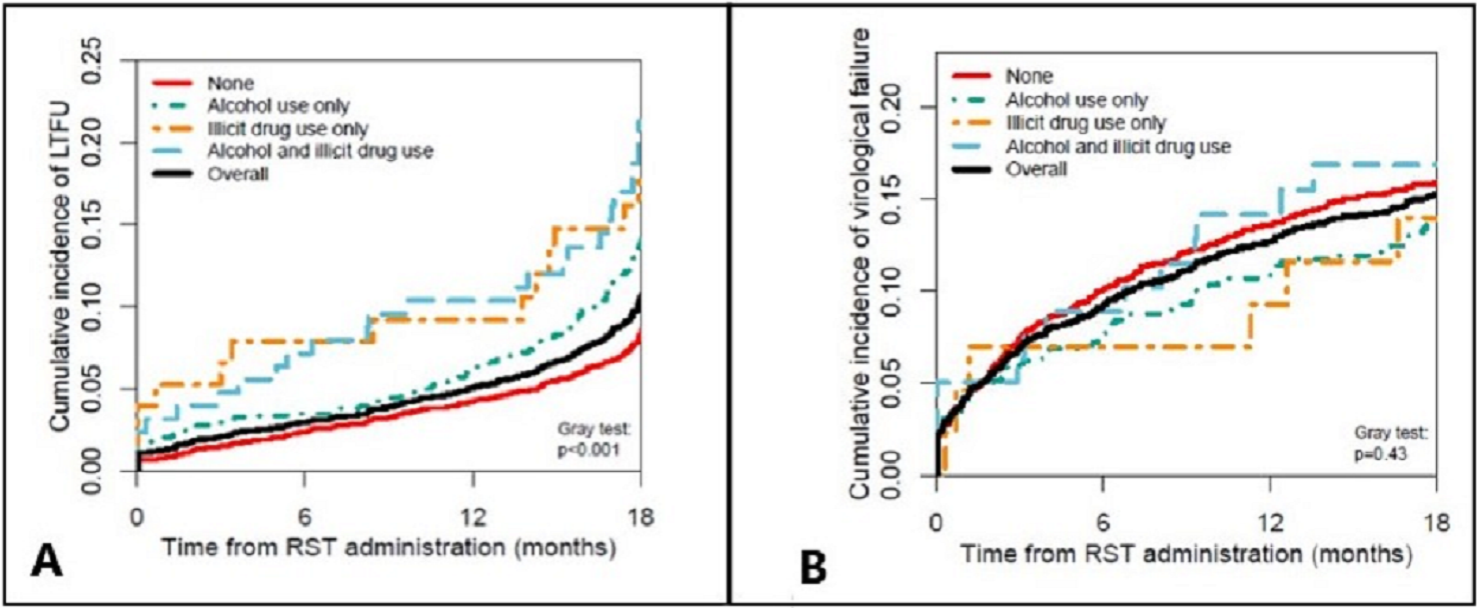
Cumulative incidences of loss to follow up (A) and virologic failure (B) in CCASAnet, stratified by alcohol and substance use, 2014–2015. LTFU = Lost to Follow Up; RST = Rapid Screening Tool.

During the 18 months following RST administration, the overall cumulative incidence of LTFU was 10% (95% CI 9–11%): 8% (95% CI 7–9%) among individuals who did not report alcohol or NIDU, 13% (95% CI 11–15%) among individuals who reported only alcohol use, 16% (95% CI 9–27%) among NIDU only, and 19% (95% CI 13–27%) among those who reported both. Fig 1A shows the cumulative incidence of LTFU over time based on alcohol and NIDU. Alcohol use was associated with an increased hazard of LTFU (adjusted hazard ratio [aHR] = 1.22, 95% CI 1.02–1.45), as was NIDU (aHR = 1.34, 95%CI 1.00–1.80); no evidence of an interaction was found (p = 0.57). In addition, men and individuals who had been on ART for a longer time also had increased LTFU risk (Table 3). Results were similar when self-reported adherence was included in the model.

**Table 3:** Cox regression models to estimate factors associated with loss to follow up after RST administration, not controlling and controlling for adherence at CCASAnet sites, 2012–2015 (n = 3604) ^a^^,^^b^^,^^c^.

	Model omitting adherence		Model including adherence	
	aHR (95% CI)	p-value	aHR (95% CI)	p-value
**Primary Exposures**				
Alcohol use	1.22 (1.02, 1.45)	0.028	1.20 (1.01, 1.43)	0.044
NIDU	1.34 (1.00, 1.80)	0.051	1.32 (0.98, 1.78)	0.070
**Demographic covariates**				
Age		0.11		0.10
20 years	0.71 (0.48, 1.04)		0.71 (0.48, 1.04)	
30 years (ref)	1		1	
40 years	1.05 (0.87, 1.27)		1.05 (0.87, 1.27)	
50 years	0.85 (0.66, 1.09)		0.85 (0.66, 1.09)	
Male (vs. Female)	1.32 (1.00, 1.74)	0.047	1.32 (1.00, 1.74)	0.048
HIV transmission mode		0.081		0.090
Heterosexual (ref)	1		1	
Homosexual	1.17 (0.92, 1.49)		1.17 (0.92, 1.49)	
IDU	0.78 (0.32, 1.92)		0.78 (0.31, 1.91)	
Other/unknown	1.51 (1.09, 2.09)		1.49 (1.08, 2.07)	
**Clinical covariates**				
Pre-cART AIDS	1.08 (0.84, 1.40)	0.54	1.09 (0.84, 1.41)	0.52
CD4 at cART initiation (per 100 cells/mm^3^)	0.99 (0.91, 1.07)	0.74	0.99 (0.91, 1.07)	0.73
CD4 at RST administration (per 100 cells/mm^3^)	0.96 (0.93, 1.00)	0.058	0.97 (0.93, 1.00)	0.071
Time on cART (10 vs. 2 years)	2.01 (1.71, 2.37)	< 0.001	2.01 (1.71, 2.36)	< 0.001
Missed cART dose(s) in 7-day recall	-	-	1.09 (0.89, 1.32)	0.42

a] LTFU is measured by > 365 days since last contact.

b] There was some evidence of non-linearity for age with the log-odds of retention (p = 0.046 with and p = 0.048 without adherence).

c] There is little evidence of an interaction between alcohol and drug use on the hazard of LTFU (p = 0.57).

Adjusting for adherence, there is little evidence of an interaction between alcohol and drug use on the hazard of LTFU (p = 0.55). Interaction terms were excluded from the models summarized above. RST = Rapid Screening Tool; NIDU = noninjected drug use.

There were 1901 individuals (53%) with at least one HIV RNA measure after RST administration. Among these patients, Fig 1B shows that there was little difference in the cumulative incidence of VF between those with no substance use, alcohol use, NIDU, or both. The overall cumulative incidence of VF after 540 days from RST was 15% (95% CI 14–17%): 16% (95% CI 14–18%) among individuals who did not report alcohol or NIDU, 14% (95% CI 11–17%) among individuals who reported only alcohol use, 14% (95% CI 6–29%) among NIDU only, and 17% (95% CI 10–27%) among those who reported both. Alcohol use (aHR = 1.07, 95% IC 0.84–1.36) and NIDU (aHR = 1.40, 95% CI 0.92–2.12) were not associated with VF in the Cox regression model. In contrast, younger age (e.g., for 20- vs. 30-year-olds, aHR = 1.31, 95% CI 1.17–1.47), CD4 at cART initiation (aHR = 1.69, 95% CI 1.12–2.56 for 300 vs. 75 cells/mm^3^), CD4 at RST administration (aHR = 0.32, 95% CI 0.23–0.45 for 700 vs. 350 cells/mm^3^), and longer time on cART (aHR = 1.52, 95% CI 1.21–1.92 for 10 vs. 2 years) were associated with VF. In the model including adherence, reporting missing 2 or more cART doses in the 7-day recall period increased the risk of VF (aHR = 1.77, 95% CI 1.39 vs. 2.25), but there were no statistically significant changes to the other variables included in the model, as can be seen in Table 4.

**Table 4:** Cox regression model to estimate factors associated with virologic failure following RST administration at CCASAnet sites, 2012–2015, (n = 1901)^a^^,^^b^^,^^c^.

	Model omitting adherence		Model including adherence	
	aHR (95% CI)	p-value	aHR (95% CI)	p-value
**Primary Exposures**				
Alcohol use	1.07 (0.84, 1.36)	0.58	1.00 (0.78, 1.27)	0.99
NIDU	1.40 (0.92, 2.12)	0.11	1.35 (0.89, 2.06)	0.16
**Demographic covariates**				
Age		< 0.001		< 0.001
20 years	1.31 (1.17, 1.47)		1.31 (1.17, 1.46)	
30 years (ref)	1		1	
40 years	0.76 (0.68, 0.85)		0.76 (0.68, 0.86)	
50 years	0.58 (0.46, 0.72)		0.58 (0.47, 0.73)	
Male (vs. Female)	0.95 (0.72, 1.25)	0.70	0.98 (0.74, 1.29)	0.88
HIV transmission mode		0.055		0.061
Heterosexual (ref)	1		1	
Homosexual	1.46 (1.09, 1.96)		1.44 (1.07, 1.92)	
IDU	0.93 (0.33, 2.60)		0.80 (0.28, 2.24)	
Other/unknown	1.47 (0.98, 2.21)		1.44 (0.96, 2.16)	
**Clinical covariates**				
Pre-cART AIDS	1.12 (0.75, 1.68)	0.57	1.18 (0.80, 1.75)	0.39
CD4 at cART initiation (300 vs. 75 cells/mm^3^)	1.69 (1.12, 2.56)	0.002	1.72 (1.14, 2.58)	0.001
CD4 at RST administration (700 vs. 350 cells/mm^3^)	0.32 (0.23, 0.45)	< 0.001	0.32 (0.23, 0.45)	< 0.001
Time on cART (10 vs. 2 years)	1.52 (1.21, 1.92)	0.002	1.51 (1.19, 1.90)	0.002
Missed cART dose(s) in 7-day recall	-		1.77 (1.39, 2.25)	< 0.001

a] Virological failure was (1) two consecutive values of more than 50 HIV RNA copies per mL; (2) a single measurement of more than 1000 HIV RNA copies per mL.

b] There was some evidence of non-linearity for three continuous covariates with the log-odds of retention; they are CD4 at cART (p = 0.28 with and p = 0.27 without adherence), CD4 at RST (p < 0.001 and p < 0.001), and time from cART initiation (p = 0.073 and p = 0.057.

c] There is little evidence of an interaction between alcohol and drug use on the hazard of viral failure (p = 0.30).

Adjusting for adherence, there is little evidence of an interaction between alcohol and drug use on the hazard of viral failure (p = 0.27). Interaction terms were excluded from the models summarized above. RST = Rapid Screening Tool; NIDU = noninjected drug use.

A total of 61 deaths were recorded during the follow up period: 50 among non-users, 10 among alcohol users only, none among NIDU only, and one among users of both alcohol and NIDU.

Regarding substance use screening and referral to treatment, most of the sites provide screening for substance use (n = 6/7) and refer individuals with substance use disorders (SUD) for treatment into the same clinic or the same health facility (n = 4/7; see S2 Table).

## Discussion

In this longitudinal study conducted in six Latin American countries among individuals under care for HIV, we observed that alcohol and NIDU in a 7-day recall period were associated with an increased hazard of LTFU. The cumulative incidence of LTFU in 18 months was 10% overall but higher among alcohol and NIDU (19%) than non-users (8%). Although the rate of non-retention also tended to be higher among alcohol users and NIDU, ranging from 16% among those reporting no use to 31% among individuals reporting both alcohol and NIDU, these associations were not statistically significant after controlling for other variables. No significant associations were found with VF, which had an incidence ranging from 14% to 17% over 18 months across all alcohol/NIDU categories.

It is hard to compare these results with other cohorts given methodological differences for substance use and cascade definitions, as well as different epidemiological profiles for alcohol and NIDU. Results are similar to those found in Switzerland, Japan, and Colombia, where NIDU increased the hazard of LTFU [23–26], but contrary to a regional longitudinal study [27]. LTFU in HIV care is decreasing in many countries [28], but higher rates may still be associated with structural deficiencies in care and patient tracking systems [27]. It is possible that the higher LTFU found among alcohol and NIDU reflects the natural history of substance use disorders (SUD). SUD are chronic and relapsing brain conditions [29], where individuals usually have many treatment gaps and require long-term, and multiple interventions until they achieve remission [30]. Another possibility for the higher risk of LTFU is a lack of SUD treatment. There is a wide range of behavioral and pharmacological treatments available for SUD [31,32]. However, even in developed countries, for instance, less than 50% of individuals with alcohol use disorders are diagnosed [33] and less than 15% receive any treatment during their lifetime [34]. It is noteworthy that this study specifically assessed current substance use, thus further clinical investigation would be necessary for accurately predicting SUD and treatment needs of individuals who screen positive.

It is possible that some substance users who were LTFU were actually dead. A review from 2009 investigating mortality among individuals LTFU after starting ART in resource-limited settings found that approximately 40% were dead, and that site-specific mortality rates were inversely associated with the percentage of patients LTFU [35]. We do not suspect mortality rates to be so high among those LTFU in our Latin American setting, as previously discussed by Carriquiry et al. [22]. In addition, most of our sites review national death registries to trace patients LTFU for mortality. With that said, under-reporting of mortality among those LTFU could still be an issue. In Russia and in the Veterans Aging Cohort Study, hazardous alcohol use increased overall mortality among HIV-infected substance users [36, 37], and substance users lost to follow up may have a higher chance of being dead than non-users. There was insufficient follow-up in our study to investigate any association between substance use and mortality; additional studies with longer follow up periods are necessary.

Neither alcohol nor NIDU was statistically associated with retention in care in our primary analysis, as found in another study [38]. However, when the number of drinks was included in the analysis (instead of any alcohol use), a larger number of drinks increased the chance of not being retained in care. This is in agreement with findings from Monroe et al. regarding heavy alcohol consumption [9]. Unfortunately, we did not screen for binge drinking (i.e, the use of five or more alcoholic drinks on a single occasion [39], which has been previously associated with non-adherence to medications and missing visits [9,40]. This, and the fact that the majority of people who drink do not have alcohol use disorders [16], highlights the need for a more detailed evaluation of alcohol use to fully disentangle the possible associations of alcohol use and HIV cascade outcomes.

The use of alcohol or NIDU was not associated with VF, suggesting that once substance users are in care and adherent to ART, they may achieve viral suppression as well as non-users. It should be noted that viral load data were only available on about half of our patients, which limits the strength of this finding. A previous CCASAnet study estimated CD4 and VL monitoring were performed at approximately 62% of the level recommended by national guidelines [41], which is similar to what we observed here. In addition, individuals who were LTFU or not retained in care, were less likely to have VL measurements, possibly impacting results. The literature is contradictory regarding the effect of NIDU on VF [42], and this conflict may be related to a lack of detail in the type, route, severity, and pattern of substance use. In the Swiss cohort, for example, only injecting drug use was associated with VF, but NIDU was not [23]. There is more evidence that substance use indirectly decreases viral suppression through decreasing adherence [19,43], and options to increase it either by treating substance use or by providing directly administered ART [8] are being investigated. We therefore acknowledge that, by grouping together all substances, we may have overlooked the effect of specific substance use on retention, LTFU, or VF.

This study has some additional limitations that should be noted. The first is that the study cohort may not represent the entire HIV-infected population of the countries. Although UNAIDS estimate that 80% of PLWHA are aware of their status in Latin America[44], to the best of our knowledge this estimate is not available for substance users in the region. As we included only individuals in HIV care services, they were all aware of their diagnosis, and thus we are not able to study the first stage of the HIV cascade. It is well known that substance users are a hard-to-reach, stigmatized population that may be underrepresented in cohort studies because of poorer access to care. This is not an issue specific to Latin America, as discussed by Volkow and Montainer [45], who advocate for a comprehensive "seek-test-treat-and-retain" approach for decreasing the HIV epidemic among substance users. Second, although the RST was sensitive enough to detect LTFU in the present study, and previously, adherence and the effect of cocaine on viral load [19,46], it is not a tool for providing SUD diagnosis, and further evaluation on how SUD affects the HIV cascade is necessary. Finally, there are unmeasured factors that may have affected the HIV cascade. In a small exploratory study, it was observed that food insecurity, financial instability, and housing instability were considered barriers to retention in care, for example [47]. Another study observed that low income was associated with not being virally suppressed [48]. The way these systemic barriers, as well as individual behavioral and demographic characteristics, affect substance users must be better understood. This is especially relevant in countries with important social inequalities because, if one considers syndemics theory [49,50], it is possible that these multiple vulnerabilities synergistically lead to worse HIV outcomes. In addition, cultural and environmental aspects of substance use, such as social tolerance and availability, may affect alcohol and NIDU, and the ways they could influence the entire HIV cascade is mostly unknown.

In conclusion, to the best of our knowledge, this is the first study evaluating the association of alcohol and NIDU with HIV cascade of care outcomes in Latin America. Our results emphasize the need for routine screening and targeted interventions to keep alcohol users and NIDU in care and on ART.

## Supporting information

S1 Table Logistic Regression: Factors associated with not being retained in care^a^—Secondary Analysis. ^a^ Adjusted for the same group of covariates as the main retention analyses including any illicit drug use. There was little evidence that the number of drinks was non-linear with the log-odds of retention; however, we also model this exposure as categorical. There was little evidence of interaction between alcohol and drug use in Models 1 and 2. a] Retention is measured by 2 or more visits at least 90 days apart during the period of interest (May 1, 2014 to May 1, 2015).
(DOCX)

S2 Table Site level characteristics regarding substance use education, screening, and referral to treatment (IeDEA Site Assessment 2.0*). *Duda SN, Farr AM, Lindegren M Lou, Blevins M, Wester CW, Wools-Kaloustian K, et al. Characteristics and comprehensiveness of adult HIV care and treatment programmes in Asia-Pacific, sub-Saharan Africa and the Americas: Results of a site assessment conducted by the International epidemiologic Databases to Evaluate AIDS (IeDEA) Collaborati. J Int AIDS Soc. 2014;17(1):1–13.
(DOCX)
