# Supplementary material for: Is substance use associated with HIV cascade outcomes in Latin America?
Source: PLoS One. 2018 Mar 15;13(3):e0194228. doi: 10.1371/journal.pone.0194228 (PMC5854364; doi:10.1371/journal.pone.0194228)
Supplement: S1 Table — a Adjusted for the same group of covariates as the main retention analyses including any illicit drug use. There was little evidence that the number of drinks was non-linear with the log-odds of retention; however, we also model this exposure as categorical. There was little evidence of interaction between alcohol and drug use in Models 1 and 2. a] Retention is measured by 2 or more visits at least 90 days apart during the period of interest (May 1, 2014 to May 1, 2015). (DOCX) [file pone.0194228.s001.docx]

**Supporting Table 1.** Logistic Regression: Factors associated with not being retained in care^a^ - Secondary Analysis

|  | Omit adherence |  | Include adherence | |
| --- | --- | --- | --- | --- |
|  | aOR (95% CI) | p-value | aOR (95% CI) | p-value |
| **Model 1:** |  |  |  |  |
| Alcohol use (per 7 drinks) | 1.19 (1.08, 1.30) | < 0.001 | 1.18 (1.07, 1.29) | < 0.001 |
| **Model 2:** |  |  |  |  |
| Alcohol use |  | 0.009 |  | 0.010 |
| None (ref) | 1 |  | 1 |  |
| 1-7 drinks | 1.11 (0.88, 1.39) |  | 1.07 (0.84, 1.35) |  |
| 8-14 drinks | 0.66 (0.37, 1.17) |  | 0.60 (0.33, 1.08) |  |
| > 14 drinks | 2.54 (1.35, 4.78) |  | 2.43 (1.29, 4.59) |  |

^a^ Adjusted for the same group of covariates as the main retention analyses including any illicit drug use. There was little evidence that the number of drinks was non-linear with the log-odds of retention; however, we also model this exposure as categorical. There was little evidence of interaction between alcohol and drug use in Models 1 and 2. a] Retention is measured by 2 or more visits at least 90 days apart during the period of interest (May 1, 2014 to May 1, 2015).
