# Supplementary material for: Is substance use associated with HIV cascade outcomes in Latin America?
Source: PLoS One. 2018 Mar 15;13(3):e0194228. doi: 10.1371/journal.pone.0194228 (PMC5854364; doi:10.1371/journal.pone.0194228)
Supplement: S2 Table — *Duda SN, Farr AM, Lindegren M Lou, Blevins M, Wester CW, Wools-Kaloustian K, et al. Characteristics and comprehensiveness of adult HIV care and treatment programmes in Asia-Pacific, sub-Saharan Africa and the Americas: Results of a site assessment conducted by the International epidemiologic Databases to Evaluate AIDS (IeDEA) Collaborati. J Int AIDS Soc. 2014;17(1):1–13. (DOCX) [file pone.0194228.s002.docx]

**Supporting Table 2.** Site level characteristics regarding substance use education, screening, and referral to treatment (IeDEA Site Assessment 2.0*)

| Country | Facility | Education on high-risk substance-use behaviors and harm reduction practices | Screening for drug and alcohol use/abuse | Referral for substance abuse treatment |
| --- | --- | --- | --- | --- |
| Argentina | Hospital Fernández | In the same Health Facility | In the same Health Facility | Provided in the Clinic |
| Brazil | INI Evandro Chagas | Not available | Provided in the Clinic | Only offsite (at distance) |
| Chile | Fundacion Arriaran | Provided in the Clinic | Provided in the Clinic | In the same Health Facility (but not at this clinic) |
| Honduras | Hospital Escuela | In the same Health Facility (but not at this clinic) | Provided in the Clinic | In the same Health Facility (but not at this clinic) |
| Honduras | Instituto Hondureno de Seguro Social | Provided in the Clinic | Provided in the Clinic | Provided in the Clinic |
| Mexico | Instituto Nacional de Ciencias Medicas y Nutricion Salvador Zubiran | Not available | Provided in the Clinic | Only offsite (at distance) |
| Peru | Instituto de Medicina Tropical Alexander von Humboldt | Not available | Not available | Not available |

*Duda SN, Farr AM, Lindegren M Lou, Blevins M, Wester CW, Wools-Kaloustian K, et al. Characteristics and comprehensiveness of adult HIV care and treatment programmes in Asia-Pacific, sub-Saharan Africa and the Americas: Results of a site assessment conducted by the International epidemiologic Databases to Evaluate AIDS (IeDEA) Collaborati. J Int AIDS Soc. 2014;17(1):1–13.
